# Effect of nutrition behavior change communication on nutrition knowledge and dietary practices of pregnant adolescents in West Arsi, Central Ethiopia: a cluster randomized controlled trial

**DOI:** 10.3389/fnut.2025.1541415

**Published:** 2025-03-27

**Authors:** Adane Tesfaye, Kefyalew Taye Belete, Dessalegn Tamiru, Tefera Belachew

**Affiliations:** ^1^Department of Nutrition and Dietetics, Faculty of Public Health, Institute of Health, Jimma University, Jimma, Ethiopia; ^2^Department of Nutrition, School of Public Health, College of Medicine and Health Science, Dilla University, Dilla, Ethiopia; ^3^Department of Public Health, College of Health Sciences and Referral Hospital, Ambo University, Ambo, Ethiopia

**Keywords:** adolescent, pregnant, nutrition intervention, Ethiopia, cluster-randomized

## Abstract

**Objective:**

This study investigated the effect of communication about nutritional behavior changes on the nutritional knowledge and dietary practices of pregnant adolescents in the West Arsi Zone, Central Ethiopia.

**Methods:**

A two-arm parallel cluster randomized controlled trial was conducted in West Arsi, Central Ethiopia, to assess a Nutritional Behavioral Change Communication (NBCC) intervention based on the Health Belief Model (HBM). Implemented by the Alliance for Development (AFD) from 16 weeks of gestation, the intervention included food preparation demonstrations and NBCC sessions for pregnant adolescents and their husbands. The study involved 207 and 219 pregnant adolescents in 14 interventions and 14 control clusters, respectively. The primary outcome was dietary practice, and the secondary outcome was nutritional knowledge. Conducted from October 15, 2022, to July 15, 2023, the intervention group attended four counseling sessions, while the control group received standard nutritional counseling. Generalized estimating equations and the difference-in-differences method were used to estimate the net treatment effect.

**Results:**

The mean age of the study participants was 17.8 ± 1.2 years, ranging from 15 to 19 years. The appropriate dietary practice rate increased by 20.3 percentage points in the intervention group and decreased by 5.6 percentage points in the control group. After controlling for possible confounders, the odds of appropriate dietary practices increased by 13% in the comparison group [AOR = 1.13; 95% CI = 1.02, 1.2], and pregnant adolescents in the intervention group had an AOR which was 3.7 times that of the comparison group in appropriate dietary practices [AOR =4.2, 95% CI = 2.6, 5.3]. The odds of good nutritional knowledge increased in both groups, however, the NBCC group had an increase 5.5 times (95%CI: 3.8, 8.1) that of the comparison group.

**Conclusion:**

NBCC through AFDs based on the HBM is an effective approach for increasing the proportion of pregnant adolescents who practice appropriately and have good nutritional knowledge.

**Clinical trial registration:**

PACTR202203696996305, Pan African Clinical Trials Registry.

## Introduction

The potential of investing in the health of children, teenagers, and women can be unlocked through improving nutrition. It is fundamental to ensure women’s nutritional status and health at all phases of life to ensure the nutritional and health of children (1, 2). One of the most significant and controllable elements affecting the health of the pregnant adolescent mother, her fetus, and her adult chronic illnesses, is thought to be good dietary practices and nutritional status. Teenagers’ long-term health trajectory may be altered by positive influences on dietary behaviors brought about by nutritional education and counseling (3, 4).

Pregnant adolescents are more willing to attend nutrition education and counseling and learn about nutrition and diet than they are before conception (5, 6). Numerous studies carried out in Ethiopia revealed that pregnant women have lower nutritional knowledge (7, 8), and the majority of studies have focused mainly on pregnant women in general, with a small amount of attention given to pregnant adolescents. All pregnant adolescents and women should receive nutritional education as a vital component of their prenatal care. Teenagers are particularly vulnerable, difficult to reach, and neglected in developing countries. In countries with limited resources, nutritional behavioral change communication (NBCC) might be an inexpensive and efficient method of preventing undernutrition in children and adolescents. The general immaturity of adolescents makes NBCC highly beneficial to them in many ways (9).

The majority of nutritional activities in Ethiopia have focused on treatment, although prevention is the most crucial measure, particularly in nations with limited resources. Successful intervention in nutrition education could be inexpensive compared to other nutritional interventions. Additionally, there are other factors at play in Ethiopia’s nutritional system outside of only the economic problem; one is lack of nutritional literacy (10). The revised National Nutrition Program (NNP) II (11) and food and nutrition policy/FNP (12) can be considered recent examples of strong policies and programmatic approaches to adolescent nutrition in Ethiopia. The overall goal of NNP II (11) is to facilitate and ignite the reduction of malnutrition to achieve zero hunger by 2030; this ambitious goal requires community-based interventions such as the NBCC. Ethiopia has made some headway toward reducing undernutrition among pregnant women, but this practice has been implemented too slowly, especially for adolescent health and nutritional issues (13). Despite continued efforts to address malnutrition, there has been a minimal reduction in the prevalence of stunting. The national prevalence of stunting in under five children according to the latest EDHS is 36.81%, whereas the prevalence of wasting is 7%, and the prevalence of underweight is 21% (14). Ethiopia is off-track to reach the United Nations sustainable development goals of ending child malnutrition by 2030 (15).

Nutritional knowledge is very important for safeguarding that pregnant adolescent receives appropriate nutrition and keeps good health (16). A lack of nutritional knowledge might lead to malnutrition and poor health (17). One of the components of adopting a healthier nutritional practice is having knowledge about nutrition and health, which is indicative of dietary habit modification. Pregnant adolescents are therefore expected to possess the knowledge essential to maintain optimum health and nutrition during pregnancy (8).

AFDs (alliances for development), formerly known as women or health development armies (WDAs), are community health volunteers; they are leaders of 30 households, and under them, there are one to five household networks. One WDA consists of six separate one-to-five connections (18). The leader will be a woman who is knowledgeable about all the 16-health extension package (HEP) programs and practices them all (19, 20). AFDs in Ethiopia have a considerable impact on reducing maternal mortality and increasing the use of child immunization services according to a systematic review of the available data (21), and this success can be extended to nutrition programs; moreover, employing AFDs might provide a feasible sustainable solution; therefore, this study investigated the effect of the NBCC delivered by AFDs on the nutritional knowledge and dietary practices of pregnant adolescents. Observational studies on the outcome of teenage pregnancy (22–24) and studies comparing the outcomes of pregnant adolescents and pregnant women (25–27) have been conducted and revealed greater risk of unfavorable feto-maternal outcomes among pregnant adolescents than among pregnant women; however, most of these studies involve only cross-sectional studies, and they are institutional-based studies. Although the diet of pregnant mothers has been thoroughly examined, pregnant adolescents have received little attention. There is a gap in community-based interventional studies examining the impact of NBCC interventions on nutritional knowledge and dietary practices among pregnant adolescents; therefore, the aim of this study was to explore the effect of nutritional behavioral change communication interventions through AFDs on the knowledge and dietary practices of pregnant adolescents in the West Arsi Zone, Central Ethiopia.

## Methods

### Study design, setting and participants

This one-year two-arm parallel-design cluster randomized controlled community trial was conducted in the West Arsi Zone, Oromia regional state, Ethiopia, 250 km from Addis Ababa. Clusters (Kebeles), the smallest administrative unit in Ethiopia, were used as the randomization unit. The zone, spanning 12,556 sq. km, includes 16 districts (13 rural and 3 urban) with a population of 2,929,894 in mid-2022. Its climate comprises 45.5% highland, 39.6% midland, and 14.9% lowland, and agriculture is the primary livelihood. Conducted from October 15, 2022, to July 15, 2023, the study enrolled pregnant adolescents before 16 weeks of gestation, excluding those who declined consent.

### Sample size determination and sampling technique

We used G*Power 3.0.10 to calculate the sample size. The following assumptions were used to calculate the required sample size (28): a level of significance of 0.05, 80% power, an effect size of 0.37, and for the difference between two independent means (two groups), intracluster correlation (ICC) = 0.03 (based on a similar published study) (29). A design effect of approximately 2 was taken, DE = 1 + (m-1) ICC, where m = Average cluster size, assumed number of observations per cluster was 32; m = 32, ICC = 0.03, DE ≈ 2, and a 10% loss to follow-up was taken into account. The total sample size was 488. Therefore, 244 pregnant adolescents in the intervention group and 244 pregnant adolescents in the control group were included. There were 28 clusters. A cluster sampling technique was used in this study.

### Recruitment, randomization and intervention allocation

From 16 districts in the zone, five had nutrition education intervention; therefore, they were excluded from the study. From the 11 eligible districts, four districts, namely, the Dodola Rural District, Adaba District, Gedeb Hasasa District and Siraro District, were selected using simple random sampling (SRS). Then, samples of nonadjacent kebeles (clusters) were selected from the four districts using SRS. Based on the proportional size allocation and considering the equal allocation of clusters in the intervention and control groups, six clusters from both the Dodola Rural district and the Adaba district and eight clusters from both the Gedeb Hasasa district and the Siraro district were taken. The lottery (SRS) method was used to allocate the intervention and control clusters. We employed cluster randomization in this study. The Consolidated Standards of Reporting Trials (CONSORT) guidelines were used for reporting the results.

Pregnant teenagers in the same cluster had a high likelihood of communicating and discussing the intervention messages; therefore, a cluster randomized trial was utilized to prevent message contamination. All pregnant teenagers who met the criteria in one cluster were enrolled in the same arm (either the intervention or control arm) to prevent information contamination. Buffer zones (nonselected clusters) were also placed between the intervention and control clusters (30, 31).

Eligible pregnant adolescents were screened through a house-to-house survey by asking about the first date of their last menstrual period and confirming pregnancy via a pregnancy test. All eligible pregnant adolescents were included in the study. The cluster was randomly assigned, and study participants were screened and enrolled by nurses working in particular districts. Stratified randomization was used, clusters were randomly allocated to study arms within different districts.

### Intervention

Community-based nutritional behavioral change communication intervention (NBCC) using the HBM (health belief model) was the intervention package for this study. This study was adapted from the recommendations of the World Health Organization (WHO), Federal Ministry of Health of Ethiopia, the blended training module on nutritional counseling by EFDRE/MOH, and similar interventional studies (32–36). Moreover, the formative assessment at the beginning of the project guided the development of the intervention tools; husbands of the pregnant adolescents were included in the NBCC, and food preparation demonstrations were included in the intervention. The intervention tools included (1) a training guide for nutrition educators, (2) a manual for nutrition educators, (3) leaflets with the core messages for the pregnant adolescent and families, and (4) counseling checklist cards.

For 1 week, the intervention procedure was pilot tested in a context comparable to the research location, and changes were made in response to the pilot testing results. Counseling was given on the basis of the MDD-W (Minimum Dietary Diversity for Women), the benefits of achieving the MDD-W and the consequences of adequate intake. The consumption of diversified food during pregnancy enables the adequate intake of important micronutrients. Inadequate dietary intake during pregnancy is the major determinant factor in the risk of pregnancy in low-birth-weight infants and is a cause of different unfavorable pregnancy outcomes. The main points of the counseling manual were increasing meal frequency (the number of times a participant take a meal during a day) and portion size as gestational age increased and eating a variety of foods, particularly those high in iron-rich foods, animal products, fruits, and vegetables. The counseling guide’s primary components also included advice on the use of iodized salt and iron/folic acid supplements. Reduced workload, day rest, the use of impregnated bed nets, and the use of medical services were additional messages of the primary contents.

The intervention was framed with the health belief model (HBM), which is a theoretical model that can be used as a guiding framework for health behavior interventions. The HBM theorizes that a person’s perceptions and beliefs about a health behavior can directly influence their probability of adopting it (37). The HBM suggests that individuals perform an internal assessment of the benefits of changing their behavior and decide whether to act. The model identifies different aspects of this assessment: (1) perceived susceptibility, (2) perceived severity, (3) perceived benefits of behavior change, (4) perceived barriers to taking action and (5) self-efficacy (38). During NBCC, it was also emphasized how poor nutritional intake might have negative effects and how susceptible pregnant adolescents are to undernutrition. The NBCC manual also covered the advantages of consuming enough meals that are varied and the obstacles to eating a balanced diet. Each pregnant adolescent in the intervention group attended four counseling sessions throughout her entire pregnancy. Home visits were used to provide personalized trimester-based NBCC. During counseling, the AFDs (counselors) employed a client-centered approach to pinpoint food habits and unique nutritional requirements. Counselors considered the needs of the pregnant adolescent, their household income, and any gaps they had found before letting the adolescent select advice that was readily available, palatable, and cheap in their area. A GALIDRAA step to counseling (Greet, Ask, Listen, Identify, Discuss, Repeat, Agree, and Appoint) was used (39).

Each counseling session for the NBCC lasted 45 to 60 min and was conducted using counseling guide cards that contained the essential information. Before 16 weeks of pregnancy, the first consultation focused on the fundamentals of nutrition, food groups, choosing foods that provide a balanced diet, demonstrating how to prepare meals, frequency of meals, and the use of iodized salt. The second and third counseling sessions, which covered the entirety of the counseling manual, were provided throughout the second trimester of pregnancy. The final counseling, which focuses on weight gain and incorporates all of the module’s important messages, was provided based on the gaps that were discovered during the previous trimesters of pregnancy.

Leaflets with the core messages in Afan Oromo and Amharic (local languages) and appropriate pictures were prepared and delivered to each pregnant adolescent in the intervention arm. For pregnant adolescents who could not read, anyone at home or in the neighborhood who can read was requested to help by reading to the adolescent and other family members.

Fourteen AFDs were used as counselors and were closely supervised by 4 BSc nurses; counselors were selected by health extension workers based on their previous experience in public health services. The counselor and supervisor had 1 week of intensive training that included role-playing and fieldwork utilizing the training manual. Additionally, three additional days of training were provided for the supervisors and counselors after the intervention had been in place for approximately 2 months to ensure that the providers continued to follow the standardized practices. Dietary practice was the primary outcome, and nutritional knowledge was the secondary outcome of this trial.

Cooking demonstration: Cooking demonstrations were offered, focusing on teaching pregnant adolescents how to prepare foods that are balanced, maintain nutrient retention, prevent nutrient loss and ensure food safety. Cooking demonstrations were performed two times on a group basis by experienced nurses at health posts. The diets in the communities participating in the study were predominantly plant-based and thus contained a high content of phytate and polyphenols that inhibit the absorption of iron, zinc and calcium. Thus, the pregnant adolescent demonstrated food processing that enables increased absorption of nutrients (40).

### Intervention fidelity

Based on the best practice recommendations developed by the National Institutes of Health Behavioral Change Consortium, criteria were constructed to evaluate the intervention’s integrity (9). Checklists evaluating the intervention design, counselor training, counseling process, reception of the intervention, and application of the skills learned from the intervention were included in the study (41). We chose nonadjacent clusters to avoid information contamination. To equalize the variations, the intervention and control groups each received an equal number of clusters from each district.

Prior to the start of the experiment, the intervention method was pretested. Additionally, to standardize the procedure, each pregnant adolescent in the intervention group had the same number and frequency of counseling sessions, and the durations of contact within an intervention group were comparable. Using a training manual, role-playing, and simulated counseling sessions, counselor training was provided. Pre-and posttraining examinations as well as a practical evaluation were used to gage the knowledge and abilities of the counselors. Randomly chosen counseling sessions were chosen for process evaluation, and one process evaluator examined all of the selected sessions. Employing a “yes/no” rating scale, the process observer evaluated the educator on factors including employing a counseling guide, providing the entire topic, length and frequency of counseling, preparation, accuracy, and the capacity to react to inquiries in a suitable manner (82). The pregnant adolescents’ knowledge of diet during pregnancy was evaluated through interviews through checklists about their understanding of the main points of the intervention. The checklist was applied to a demonstration of food preparation and consumption to evaluate the implementation of the intervention.

Due to the nature of the intervention, participant allocation concealment was not practical. The groups were labeled with a unique number until analysis was finished, which also served to blind the data input clerk. The lead investigator and counseling supervisors oversaw the counseling process.

### Data collection procedure and measurements

The primary outcome of this study was dietary practice measured within 24 h. dietary recall method, while the secondary outcome was nutritional knowledge. We used a pretested structured questionnaire to collect the data. The questionnaire included questions on sociodemographic factors, health service usage, dietary categories, meal frequency, and Household Insecurity Access Scale (HFIAS). Two master holders in public health (MPH) holders and six clinical nurses worked as supervisors and data collectors, respectively. Three female laboratory technicians performed the pregnancy tests. At the participants’ homes, the data collectors did face-to-face interviews to fill the questionnaire. In order to preserve the teenager’s privacy as much as possible, other family members were not permitted entry to the site of the interviews.

Dietary intake was calculated using 24-h recalls in accordance with the Food and Nutrition Technical Assistance (FANTA) III recommendation from the Food and Agricultural Organization (FAO) and the United States Agency for International Development (USAID). The goal was to determine whether pregnant teenagers’ diets were diverse (42). According to the recommendations, there are 10 food groups, including grains, plantains, white roots and tuber pulses (peas, beans, and lentils); dairy; meat; nuts and seeds; eggs; dark green leafy vegetables; poultry and fish; other vegetables and other fruits and vegetables rich in vitamin A. A pregnant adolescent was deemed to consume a sufficiently diverse diet if she had consumed at least five of the foods on the lists in the 24 h prior to the data collection period (42, 43). Participants were asked to recall every meal they had consumed during the preceding 24 h, both inside and outside of the home. In addition, the participants were prompted to recall any between-meal snacks they may have had. Food items were scored “1″ for consumption over the reference period and “0″ for nonconsumption.

The HFIAS guidelines (44) were used to assess food security. Twenty-seven questions on the Household Food Insecurity Access Scale were used to evaluate the households’ level of food security. Prior to this, the questions were approved for use in underdeveloped nations (45). Food-secure households that experienced fewer than the first two food insecurity indicators, households that experienced between 2 and 10, 11 to 17, and more than 17 food insecurity indicators were considered mildly, moderately, and severely food insecure, respectively.

Principal component analysis (PCA) was used to generate a wealth index by considering fixed assets. Thirty-five variables were used, and some of the variables included the availability of a water source, type of living house, presence of a latrine, bank account, agricultural ownership, livestock, and household assets (46–50). The responses of all no dummy variables were dichotomized and coded as “1” for the household possessing the asset and “0” for the rest, based on the WHO/UNICEF definition. Household income was classified into quintiles (46).

The assumptions of PCA were checked, and the correlation matrix for the variables containing two or more correlations was ≥ 0.30; the Bartlett test of sphericity was used (*p* value < 0.05); variables with sampling adequacy measures less than 0.50 were removed (looking anti-image); and the overall measures of sampling adequacy were Kaiser–Meyer–Olkin (KMO) ≥ 0.5 and (47) communality > 0.5, not having the complex structure of correlation ≥ 0.40. Components that collectively explained more than 60% of the variance in the set of variables and had eigenvalues ≥ 1 (48) were used to identify variables to be included in further analyses. The economic status of the study subjects was categorized as poorest, poor, medium, rich, or richest (46).

Eight questions were used to measure the autonomy of pregnant adolescents. When a choice was made by the pregnant adolescent alone or jointly with her husband, code 1 was assigned for each question; otherwise, code 0 was assigned. The ability of pregnant adolescents to make decisions was categorized using the mean (46). Maternal knowledge of food intake during pregnancy was evaluated using 12 questions. When a response was correct, a code of 1 was issued; otherwise, a code of 0 was given. The data were collected on HBM constructs with 25 questions. Code 1 was provided for each question when participants responded yes, and code 0 was given if they responded no; the means were compared between the intervention and control groups (46).

### Operational definitions

MDD-W: MDD-W or adequate dietary diversity was achieved when the pregnant adolescent consumed food from five or more food groups on the day or within 24 h before data collection (42).

Inadequate dietary diversity: Pregnant adolescents consumed food from fewer than five food groups on the day/within 24 h/before data collection (42).

Nutrition knowledge: Pregnant adolescents who scored ≥75% on the knowledge questions were considered to have good knowledge; otherwise, they were considered to have a low knowledge score (46).

### Reliability and validity of the data collection tools

A test–retest method was used for reliability analysis of the pregnant adolescent nutritional knowledge variable, and questions were reviewed to assess whether the results were the same for all respondents. A Cronbach coefficient of 0.83 was obtained after the pretest. To ensure validity, nutrition experts from Dilla University, the Department of Nutrition, were involved in checking the questionnaire to ensure that the questions elicited the required response, and a pretest was also conducted.

### Data management and analysis

The Kobo toolbox was used to enter data, which were subsequently exported to SPSS version 25 software for analysis. A chi-square test was used to evaluate the differences in the sociodemographic parameters between the two groups. The McNemar test was used to analyze differences between pre-and postintervention dietary practices and nutritional awareness.

The difference in outcome measures (Good dietary practice and Good nutritional knowledge) between the intervention and control groups was examined using the generalized estimating equation (GEE) with a binary logit function. First, we performed correlations on all the structures, and the correlation matrix structure with the least amount of Quasi-Information was the exchangeable correlation structure; therefore, this structure was used. The clustering variables in the GEE models were participants. The impacts of various confounding factors were taken into account when fitting the model. The analysis included sociodemographic factor information, obstetric trait information, food security information, time, treatment, and time and treatment interactions. Time and treatment interactions were used to evaluate the intervention’s impact. A multivariable GEE binary logistic model was carried out to account for within-cluster correlation of measurements. Odds ratios and the associated 95% confidence intervals were calculated, and statistical significance was defined as a *p* value of 0.05 according to the multivariable GEE binary logistic model. A per-protocol analysis was performed in this study. The per-protocol analysis included all study participants who adhered to the predetermined guidelines. Therefore, in this study, pregnant adolescents who attended four education sessions and provided end-line data were included. Difference-in-differences (DID) was used; the DID analysis controls for both unobservable and observable time-invariant characteristics with time-invariant effects. In the correlation matrix analysis dietary practice was measured by number of food groups consumed and Nutrition knowledge was measured by percentage of correct answers from 12 items on nutrition knowledge questions.

### Patient and public involvement

Patients or the general public were not actively involved in the design of this study; however, the study’s objectives were disclosed to and approved by the study participants, Jimma University, and the zonal and district health offices. Additionally, we intended to disseminate the findings to the West Arsi zonal and relevant district health offices.

### Ethical considerations

Ethical approval was obtained from the Jimma University IRB/ethics committees with reference numbers JUIH/IRB/194/22 and the Oromia Regional Health Office. Each study participant received a thorough description of the study’s title, goal, protocol, and duration, as well as the potential risks and benefits, prior to providing informed consent. Each teenager provided verbal, written, and sign informed consent prior to any interview or measurement. Informed consent was obtained from the LAR (legally authorized representative) for study participants aged 18 years and younger. Participants were made aware of the publication of their anonymous comments. Informed consent was obtained from participants prior to the commencement of interviews. The researcher remained truthful to the academic and ethical requirements. Finally, the researcher kept the data in a locked file cabinet in a safe place after the completion of the study. Informed consent was obtained from both the adolescent and their husband or parents. Finally, any ethical issues that arose during this research were resolved through discussion between the researcher and JU’s IRB.

## Results

### Socio-demographic characteristics of pregnant adolescents

Among the 459 pregnant adolescents who were enrolled in this study, 426 [intervention group (IG) =207 and control group (CG) = 219] fully adhered to the protocol and were included in the analysis. At baseline, there was no significant difference in socio-demographic characteristics between the intervention and control groups (*p* > 0.05; Table 1).

**Table 1:** Sociodemographic characteristics of pregnant adolescents in the West Arsi Zone, Central Ethiopia, 2023.

Variable	Intervention group (n1 = 207) Frequency %	Control group (n2 = 219) Frequency %	*p* value
Number of clusters	14	14	
Age			
Middle adolescent	51 (24.7)	46 (21)	0.37
Late adolescent	156 (75.3)	173 (79)	
Family size			
< 5	148 (71.5)	173 (79)	
> = 5	59 (28.5)	46 (21)	0.07
Marital status			
Married	174 (84)	188 (85.8)	0.6
Unmarried/divorced	33 (16)	31 (14.2)	
Educational status			
No formal education	20 (9.6)	30 (13.6)	
Can read & write	27 (13)	26 (11.8)	0.2
Primary education	113 (54.5)	102 (46.5)	
Secondary education	35 (16.9)	52 (23.7)	
College and above	12 (5.7)	9 (4)	
Occupation			
House wife	71 (34.3)	64 (29.2)	
Student	77 (37)	86 (39.3)	0.9
Merchant	34 (16.4)	39 (17.8)	
Daily laborer	7 (3.4)	9 (41)	
Farmer	9 (4.3)	9 (41)	
Government job	9 (4.3)	12 (5.4)	
Wealth index			
Poorest	42 (20)	46 (21)	
Poor	32 (15.4)	48 (21.9)	
Medium	32 (15.4)	17 (7.7)	0.67
Rich	59 (28.5)	70 (32)	
Richest	42 (20.3)	38 (17.3)	

### Health belief model constructs for pregnant adolescents

At end-line, Pregnant adolescents in the intervention group had significantly higher scores in perceived susceptibility (*p* < 0.001), severity (*p* = 0.004), and benefits (*p* < 0.001) than the control group. There were no significant baseline differences between groups (*p* > 0.05). Some constructs in the control group also showed significant changes over time, which may be due to external influences such as routine healthcare messages or community discussions (Table 2).

**Table 2:** Comparisons of the health belief model construct scores within and between the intervention and control groups (paired *t* test and independent *t* test, respectively) among pregnant adolescents in the West Arsi Zone, Central Ethiopia, 2023.

HBM constructs	Study period	HBM constructs score of intervention group Mean(±SD)	HBM constructs score of control group Mean(±SD)	*p* value
Perceived susceptibility	Baseline	3.66 ± 0.9	3.74 ± 2	0.07
	End-line	4.22 ± 0.9	3.47 ± 1	<0.001
	P	<0.001	<0.03	
Perceived severity	Baseline	3.52 ± 1	3.34 ± 1	0.16
	End-line	4.32 ± 1	3.52 ± 1	0.024
	P	0.004	0.004	
Perceived benefits	Baseline	3.1 ± 1.2	3.0 ± 1.2	0.24
	End-line	4.7 ± 0.87	3.5 ± 1	<0.001
	P	<0.001	<0.001	
Perceived barriers	Baseline	2.45 ± 1.4	2.61 ± 1.3	0.09
	End-line	2.10 ± 1.3	2.58 ± 1.2	0.3
	P	0.02		

HBM, Health Belief Model.

### Effect of the NBCC on the nutritional knowledge and dietary practices of pregnant adolescents

Before the implementation of the trial, there was no statistically significant difference in nutritional knowledge or dietary practice between the two groups (Table 3). After the trial, pregnant adolescents in the intervention group showed significant improvements in dietary practices and the frequency of meals compared with their dietary practices before the intervention. At the end of this trial, the proportion of pregnant adolescents who had appropriate dietary practices increased by 20.3% (from 21.7 to 42%) in the intervention group. However, the number of pregnant adolescents who had appropriate dietary practices decreased by 5.6% (from 21.5 to 15.9%) in the control group. There was a statistically significant difference in the mean minimum dietary diversity score (MDD-W) and mean meal frequency between the intervention and comparison groups (*p* < 0.001). There were statistically significant differences in the mean nutritional knowledge score, MDD-W score and mean meal frequency (Table 4).

**Table 3:** Baseline nutritional knowledge and minimum dietary diversity score (MDD-W) among pregnant adolescents (independent sample *t* test) in the West Arsi Zone, Central Ethiopia, 2023.

Variable	Intervention group (n1 = 207) N (%)	Control group (n2 = 219) N (%)	*p* value
Nutrition knowledge			
Low	158 (76.3)	167 (76.3)	0.98
Good	49 (23.6)	52 (23.7)	
MDD-W			
Inadequate	162 (78.3)	172 (78.5)	0.94
Adequate	45 (21.7)	47 (21.5)	

**Table 4:** Comparisons of mean nutritional knowledge, minimum dietary diversity score (MDD-W), and meal frequency among pregnant adolescents (using independent sample *t* test) in the West Arsi Zone, Central Ethiopia, 2023.

Variable	Intervention group (n1 = 207) Mean(±SD)	Control group (n2 = 219) Mean(±SD)	*p* value
Good nutrition knowledge			
Baseline	4.7 ± 1.7	4.6 ± 1.6	
End-line	7.64 ± 2.5	4.85 ± 1.7	<0.01
MDD-W			
Baseline	4.17 ± 1	4.2 ± 1.2	
End-line	5.66 ± 1.4	4.0 ± 1.3	<0.001
Meal frequency			
Baseline	2.7 ± 0.4	2.8 ± 0.37	
End-line	3.5 ± 0.6	2.9 ± 0.43	<0.01

On the multivariable generalized estimating equations model, after adjusting for probable confounders, the odds of appropriate dietary practices increased more in the intervention group than in the comparison group [AOR = 4.2, 95% CI: 2.6–5.3]. Pregnant adolescents in the intervention group had an AOR which was 5.5 times that of the comparison group regarding nutritional knowledge (AOR = 5.5, 95% CI = 3.78–8.1; Table 5). Differences between baseline and baseline dietary practices and differences in DID between the intervention and control groups are shown in Table 6. Furthermore, the correlations of the health belief model constructs with knowledge and dietary practices are shown in Table 7. There was no statistically significant difference in the mean score of nutritional attitudes, food security and autonomy of pregnant adolescents (Table 8).

**Table 5:** GEE results of the NBCC intervention effect (exchangeable correlation structure was used).

		Beta coefficient	Standard error	95% CI	*p* value	AOR	95% CI
Good nutrition knowledge	Intercept	−1.224	0.176	−1.57, 0.87	<0.001	0.29	0.2–0.42
	time	0.074	0.042	−0.09, 0.15	0.008	1.7	1.2–1.17
	group	−1.775	0.3842	−2.5, −1.02	<0.001	0.169	0.08–0.36
	Time* treatment	2.473	0.283	1.62, 4.37	<0.001	**5.525**	3.78–8.1
MDD-W	Intercept	0.995	0.1821	1.35,29.8	<0.001	2.7	1.8,3.86
	time	0.129	0.056	0.24	0.0023	1.13	1.02–1.2
	group	−2.5	0.33	−3.14, −1.85	<0.001	0.082	0.04–0.15
	Time* treatment	1.315	0.182	0.957	<0.001	**3.72**	2.6, 5.3

Exchangeable correlation structure was used. Correlation parameter estimated for first model = 0.007382; MDD-W, minimum dietary diversity score for women, NBCC, Nutrition behavioral change communication. Correlation parameter estimated for second model (MUAC) = 0.006827. Bold value indicates highly significant results, emphasizing the strong intervention effect. *A symbol is used to indicate interaction. TimeTreatment shows the interaction term, which represents the combined effect of time and intervention treatment.

**Table 6:** Change between baseline and end line nutritional knowledge and dietary practices and differences (DIDs) between the intervention and control groups.

Variable	Study period	Intervention group	Comparison group	DID	*p* value
Good nutrition knowledge	Baseline	4.72 ± 1.7	4.6 ± 1.6	2.67 ± 1.36	<0.001
	End-line	7.64 ± 2.5	4.85 ± 1.7		
	Average change (EL-BL)	2.92 ± 1.23	0.25 ± 1.3		0.003
MDD-W Mean(±SD)	Baseline	4.17 ± 1	4.2 ± 1.2	1.69 ± 1.32	<0.001
	End-line	5.66 ± 1.4	4.0 ± 1.3		
	Average change (EL-BL)	1.49 ± 0.9	−0.2 ± 1.24		0.002
Meal frequency	Baseline	2.7 ± 0.4	2.8 ± 0.37	0.7 ± 0.84	<0.001
	End-line	3.5 ± 0.6	2.9 ± 0.43		
	Average change (EL-BL)	0.8 ± 1.2	0.1 ± 0.64		0.003

DID, difference-in-differences; EL, end line; BL, baseline; CI, confidence interval; MDD-W, minimum dietary diversity scores for women; Linear Regression Model was used for the difference in differences estimations and the t-test was used to test the statistical significance of the DID estimate.

**Table 7:** Correlations of the health belief model constructs with knowledge and dietary practices in West Arsi, Central Ethiopia, in 2023.

	Perceived barriers	Perceived severity	Perceived benefits	Perceived susceptibility	Dietary practice	Nutrition knowledge
Perceived Barriers	1					
Perceived Severity	0.078 0.107	1				
Perceived Benefits	0.068 0.164	0.869** <0.001	1			
Perceived Susceptibility	0.090 0.064	0.943** <0.001	0.917** <0.001	1		
Dietary practice	−0.12* 0.034	0.198 <0.001	0.133** <0.001	0.165** <0.001	1	
Nutrition Knowledge	−0.2 0.013	0.2** <0.001	0.274** <0.001	0.236** <0.001	0.054 0.269	1

Correlations estimated at endline. *Correlation is significant at the 0.05 level (2-tailed), **Correlation is significant at the 0.01 level (2-tailed); Good nutrition knowledge: Pregnant adolescents who scored ≥75% on the knowledge questions; Dietary practice measured by the 10 food groups; which is MDD-W, minimum dietary diversity scores for women.

**Table 8:** Comparisons of nutritional attitude, food security and autonomy of pregnant adolescents among pregnant adolescents (using independent sample *t* test) in the West Arsi Zone, Central Ethiopia, 2023.

Variable	Intervention group	Control group	*p* value
	(n1 = 207)	(n2 = 219)	
	Mean(±SD)	Mean(±SD)	
Nutritional attitude			
Baseline	7.3 ± 2.2	7.1 ± 2.5	0.38
End-line	9.6 ± 2.4	7.4 ± 2.2	
Food security			
Baseline	6.3 ± 2.8	6.1 ± 3.0	0.72
End-line	6.6 ± 2.5	6.4 ± 2.6	
Autonomy of pregnant adolescents			
Baseline	3.6 ± 1.3	3.5 ± 1.4	0.32
End-line	6.3 ± 1.9	3.9 ± 1.5	

### Prediction of dietary diversity among pregnant adolescents

The multivariable generalized estimating equation (GEE) logistic regression model revealed that having a college education or above (AOR = 4.37, 95% CI = 2.90, 5.62), being a merchant (AOR = 6.37, 95% CI = 5.20, 7.18), having a richest wealth quintile (AOR = 3.34, 95% CI = 2.27, 4.47) and good nutritional knowledge (AOR =5.47, 95% CI = 4.2, 6.29) were significantly more likely to increase dietary diversity (Table 9).

**Table 9:** Logistic regression model for multivariate generalized estimating equations predicting MDD-W among pregnant adolescents.

Variable	Category	*p* value	AOR	95% CI
Maternal age	Middle adolescent		Ref.	
	Late adolescent	0.83	1.28	(0.63–4.82)
Educational status	No formal education		Ref.	
	Can read & write	0.47	1.47	(0.27–4.36)
	Primary education	0.83	1.62	(1.20–7.87)
	Secondary education	0.27	2.72	(0.83–6.43)
	College and above	0.01	4.37	(2.90–5.62)
Occupation	Housewife		Ref.	
	Student	0.73	1.62	(1.23–7.62)
	Merchant	<0.01	6.37	(5.20–7.18)
	Daily laborer	0.92	2.30	(0.81–3.72)
	Farmer	0.27	1.85	(0.97–3.73)
	Government job	0.29	2.47	(0.62–8.68)
Wealth index	Poorest		Ref.	
	Poor	0.57	1.37	(0.49–3.45)
	Medium	0.29	2.47	(1.83–5.48)
	Rich	0.70	2.65	(0.8–4.74)
	Richest	0.03	3.34	(2.27–4.47)
Food security	Food insecure		Ref.	
	Food secured	0.62	2.37	(0.9–5.63)
End line nutrition knowledge	Good	0.02	5.47	(4.2–6.29)
	Low		Ref.	

The model was adjusted for age, education, wealth index, nutritional knowledge, and food security status. AOR, adjusted odds ratio; CI, confidence interval; SE, standard error; Ref, reference category, good nutrition knowledge Pregnant adolescents who scored ≥ 75% on the knowledge questions; correlation parameter is estimated = 0.006723.

## Discussion

This study revealed that the nutritional knowledge and dietary practices of pregnant adolescents improved after NBCC was delivered through AFDs via the HBM. The proportion of adolescents in the intervention group who had appropriate dietary practices was significantly greater than that of adolescents in the comparison group. These results persist after controlling for potential confounders.

These findings are in agreement with the findings of similar studies from West Gojam Ethiopia (34), Dessie Town Ethiopia (51), and Malawi (52), which reported significant improvements in the dietary practices of pregnant women. Improvements in nutritional knowledge and good dietary practices lead to positive outcomes, such as appropriate weight gain and improved birth outcomes, thereby preventing low birth weight still birth, IUGR (intrauterine growth restriction), premature delivery and unfavorable overall nutritional outcomes (53, 54).

The nutritional knowledge score of pregnant adolescents in this study increased significantly after NBCC, which was comparable with the findings of different studies conducted in Ethiopia (55), the Eretria (56), Malawi (52), and Iran (57). The use of the NBCC might help pregnant adolescents to have a better understanding of the severity of consequences that may result from poor dietary practices, the benefits of good dietary practices, and the level of susceptibility they have and to identify and manage barriers, all of which increase nutritional knowledge (58, 59). Although nutritional knowledge does not guarantee good dietary practices, it is a strong predisposing factor for good dietary practices.

Except for perceived barrier, all the other HBM constructs had a significant positive correlation with the nutritional knowledge and dietary practices of pregnant adolescents. This finding is consistent with the findings of previous studies reporting the successful use of nutritional counseling via HBM constructs to induce behavioral changes toward a healthy diet (34, 60–62). This might be because pregnant adolescents who attend nutrition education according to the HBM perceive that the consequences of poor diet are very severe and might also consider themselves susceptible to the consequences of poor diet. Moreover, pregnant adolescents might perceive that the benefits of consuming adequate and diversified food outweigh the barriers to accessing such food.

Meal frequency significantly improved by one meal per a day after the intervention, and a similar finding was revealed in an interventional study conducted in West Gojjam, Ethiopia (34). The present study provided evidence that the NBCC can help pregnant adolescents change their dietary pattern or meal frequency.

The Institute of Medicine recommends three meals and two or more snacks per day for optimal pregnancy outcomes (63). However, in Ethiopia, cultural dietary habits, food availability, and economic constraints may affect adherence (64). NBCC can help pregnant adolescents make informed choices using locally available, nutrient-dense foods to meet their increased energy needs.

Pregnant adolescents with better sociodemographic and economic status (educational status, occupation and wealth index) were more likely to consume a diet with adequate dietary diversity than those with lower status. This finding is consistent with the findings of similar studies conducted in urban settings in Southeast Ethiopia (61), rural southwestern Ethiopia (62), and Laikipia, Kenya (65). Similarly, Pregnant adolescents with good nutritional knowledge were more likely to achieve adequate dietary diversity than were those with low nutritional knowledge; this evidence is supported by studies conducted in the Ambo district, Ethiopia (66). Systematic reviews performed in Ethiopia (67) and Malawi (68). Nutritional knowledge helps pregnant adolescents understand the specific nutrient requirements during pregnancy and the importance of consuming a wide range of foods to meet these needs and support their own health and the growth and development of the fetus (69).

The GALIDRAA counseling technique employed in this intervention and teaching materials such as leaflets, cooking demonstration methods, use of HBM and husbands’ involvement in education sessions might have led to significant improvements in knowledge and dietary practices. In addition, the counselors used trimester-based NBCC and assessed the existing knowledge, dietary practices and socioeconomic situation of each adolescent. Then, counseling was given based on the needs of specific adolescents, which may further increase the success of the intervention. Moreover, in addition to its theoretical components, this behavioral change communication strategy featured a practical demonstration to improve food preparation and processing skills for local foods to promote consumption, absorption, and utilization of micronutrients; this could also be the other reason for significant improvement in nutritional knowledge and good dietary practices.

AFDs are model front-line or community-level actors who have obtained much experience and knowledge by closely working with health extension workers and nurses; they can provide home-to-home and health post-nutrition behavioral change communication, which also decreases the burden on health extension workers; therefore, it would be good to task shift some public health intervention packages, such as nutrition education, to AFDs. The busy schedule of health extension workers and small number of nurses is the other problem; therefore, if AFDs are well trained and incentivized and if close supportive supervision is given, they can play a crucial role in helping pregnant individuals and other parts of the population. The decentralization and devolution of nutritional and health services to the grassroots level are crucial, and these interventions can be sustainable in resource-poor settings, including Ethiopia.

There is no assurance that pregnant adolescents will be able to communicate about nutritional behavioral changes at a specific ANC appointment because there are numerous competing subjects under ANC services (70, 71); if provided in such circumstances, this communication is probably not thorough or well detailed, making it unlikely to improve nutritional literacy (72). In addition, low pregnancy attendance at ANC in Ethiopia (45) and high client/health care provider ratios in health facilities (73, 74) restrict access to interactive or individual nutrition education sessions. According to a national report (75), only 32% of pregnant individuals receive optimal antenatal care. AFDs can be used to support usual ANC nutrition education sessions with community-based nutrition interventions.

NBCC interventions that use local nutrient-dense foods are preferable over micronutrient supplements (76, 77) because they can sustainably increase dietary sufficiency of micronutrients without running the risk of antagonistic micronutrient interactions (77). Food-based approaches involving nutritional intervention, however, are not optimal if the diet is deficient in nutrients (76) or if nutrients are wasted due to poor absorption from medical conditions, including gut infections (78). To ensure micronutrient bioavailability and food hygiene, NBCC should be offered alongside other fundamental interventions, such as general health education, food preparation and processing demonstrations, and food safety training (79).

The impact of nutrition education may be impacted by food resource scarcity, which is worsened by poverty and all of its consequences, including household food insecurity, which prolongs nutritional vulnerability (80). Thus, pregnant individuals are unlikely to employ their knowledge and recommendations if the suggested foods are more difficult to obtain, such as in situations of financial hardship or seasonal production unpredictability. The effectiveness of the NBCC depends on elements such as community involvement, health system strengthening, and addressing social determinants of malnutrition. The effect of the NBCC is greater when combined with other forms of nutrition-specific and nutrition-sensitive interventions to successfully improve maternal and child nutrition (81). Future studies are needed to investigate culturally sensitive and context-specific nutritional education interventions to address the unique needs and challenges faced by pregnant adolescents and interventions to improve adherence to iron folic acid supplementation. Moreover, studies and interventions are needed to prevent adolescent pregnancy.

Limitations of the study include the following: the DID approach does not control for omitted confounders with time-varying effects. Individuals’ food intake from the previous 24 h. These data were taken into account, which may not accurately reflect their typical eating patterns. Dietary intake can change during lean or bumper seasons as well as during celebrations, none of which were taken into account in this study. The 24-h. The data do not represent the long-term dietary habits of the participants. Due to the relatively short duration of the intervention, the postintervention result might not have persisted longer.

## Conclusion

Nutritional behavioral change communication through the Alliance for development using the health belief model is an effective approach for increasing the proportion of pregnant adolescents who practice appropriate dietary practices. Pregnant adolescents in the intervention group had higher MDD-W scores, greater nutritional knowledge and greater meal frequency scores than did adolescents in the control group. Therefore, the findings of this study suggest the need for employing a model-based NBCC through community-level actors to improve the dietary knowledge and practices of pregnant adolescents.

## Data Availability

The raw data supporting the conclusions of this article will be made available by the authors, without undue reservation.

## Glossary

AFDs: Alliance for Development
ANC: Antenatal Care
AOR: Adjusted Odds Ratio
BL: Baseline
CG: Control Group
CI: Confidence Interval
CONSORT: Consolidated Standards of Reporting Trials
DID: Difference-In-Differences
EL: End Line
FANTA: Food and Nutrition Technical Assistance
FAO: Food and Agricultural Organization
FMOH: Federal Ministry of Health of Ethiopia
GALIDRAA: Greet, Ask, Listen, Identify, Discuss, Repeat, Agree, and Appoint
GEE: Generalized Estimating Equation
HBM: Health Belief Model
HEP: Health Extension Package
HFIAS: Household Food Insecurity Access Scale
ICC: Intracluster Correlation
IG: Intervention Group
IUGR: Intrauterine Growth Restriction
JUIH/IRB: Jimma University Institutional Review Board
MDD-W: Minimum Dietary Diversity for Women
MOH: Ministry of Health
MPH: Master of Public Health
NBCC: Nutritional Behavioral Change Communication
NNP: National Nutrition Program
PCA: Principal Component Analysis
SRS: Simple Random Sampling
USAID: United States Agency for International Development
WDA: Women Development Army
WHO: World Health Organization
